# Supplementary material for: Construction and validation of a novel cuproptosis-related long noncoding RNA signature for predicting the outcome of prostate cancer
Source: Front Genet. 2022 Dec 6;13:976850. doi: 10.3389/fgene.2022.976850 (PMC9763621; doi:10.3389/fgene.2022.976850)
Supplement: Supplementary file 2 [file DataSheet1.ZIP › Source data for review purpose only/Source data/25.pRRophetic/Cor.SL 0101-1.pdf]

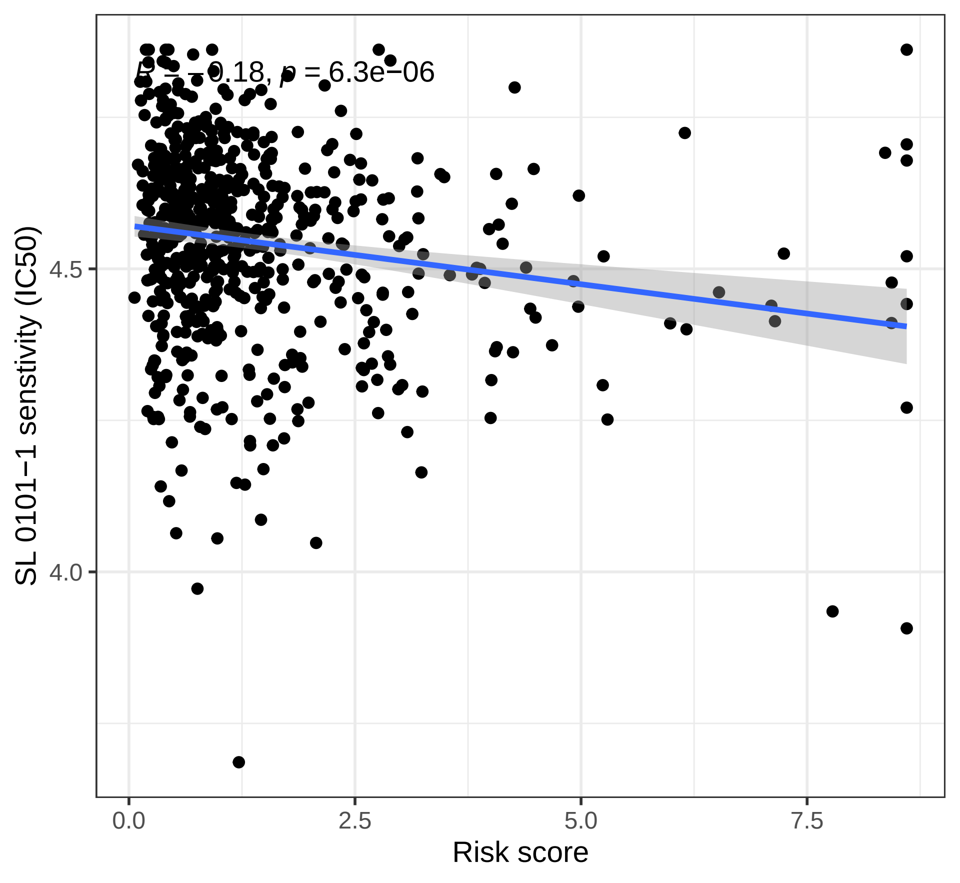

R = −0.18, p = 6.3e−06
SL 0101−1 senstivity (IC50)
4.5
4.0
0.0
2.5
5.0
7.5
Risk score
